# A rare case of pancreatic metastasis from malignant melanoma mimicking pancreatitis on ^18^F-FDG PET/CT

**DOI:** 10.1186/s43046-021-00087-z

**Published:** 2021-10-11

**Authors:** Luca Filippi, Ilaria Proietti, Orazio Schillaci, Concetta Potenza, Oreste Bagni

**Affiliations:** 1 Department of Nuclear Medicine, “Santa Maria Goretti” Hospital, Via Antonio Canova, 04100 Latina, Italy; 2 Dermatology Unit “Daniele Innocenzi”, “A. Fiorini” Hospital, Via Firenze, 1, 04019 Terracina, LT Italy; 3 Department of Biomedicine and Prevention, University Tor Vergata, Viale Oxford 81, 00133 Rome, Italy; 4 IRCCS Neuromed, 86077 Pozzilli, Italy

**Keywords:** Malignant melanoma, PET/CT, Immunotherapy, Precision medicine

Editor,

Here we report the case of a 51-year-old male who underwent surgery due to left forearm malignant melanoma (MM) in 2007. He did not perform adjuvant therapy and was submitted to monitoring. He remained disease-free until December 2020, when skin nodules appeared in the left arm, confirmed by histological examination as metastases. Reverse transcriptase-polymerase chain reaction (RT-PCR) revealed no known mutations affecting the gene encoding serine–threonine protein kinase BRAF. After having provided his written consent, he was submitted to ^18^F-fluorodeoxyglucose positron emission tomography/computed tomography (^18^F-FDG PET/CT) for restaging. ^18^F-FDG whole body PET/CT (Fig. 1) demonstrated extremely increased tracer incorporation in the cutaneous/subcutaneous metastasis in the left arm, as well as diffuse and intense ^18^F-FDG uptake in the whole pancreas, which also resulted significantly enlarged. The patient was completely asymptomatic, serum amylase and lipase levels were normal, therefore acute pancreatitis was considered an unlikely diagnosis. Magnetic resonance imaging (MRI), performed at a clinical center external respect to our facility, showed multiple confluent pancreatic lesions, hyperintense on T1-weighted and hypointense on T2-weighted images (images not available). On the basis of the agreement between PET/CT, MRI and clinical data, taking into account patien’s urgency to promptly start therapy, endoscopic ultrasound-guided fine-needle biopsy (EUS-FNB) was not performed and immunotherapy (IT) with nivolumab (480 mg I.V. q 4 weeks) was started. Three months after the start of IT, the patient underwent a follow-up PET/CT that showed complete regression of both hypermetabolic lesions in the left arm and in the pancreas, as shown in Fig. 2.

**Fig. 1:**
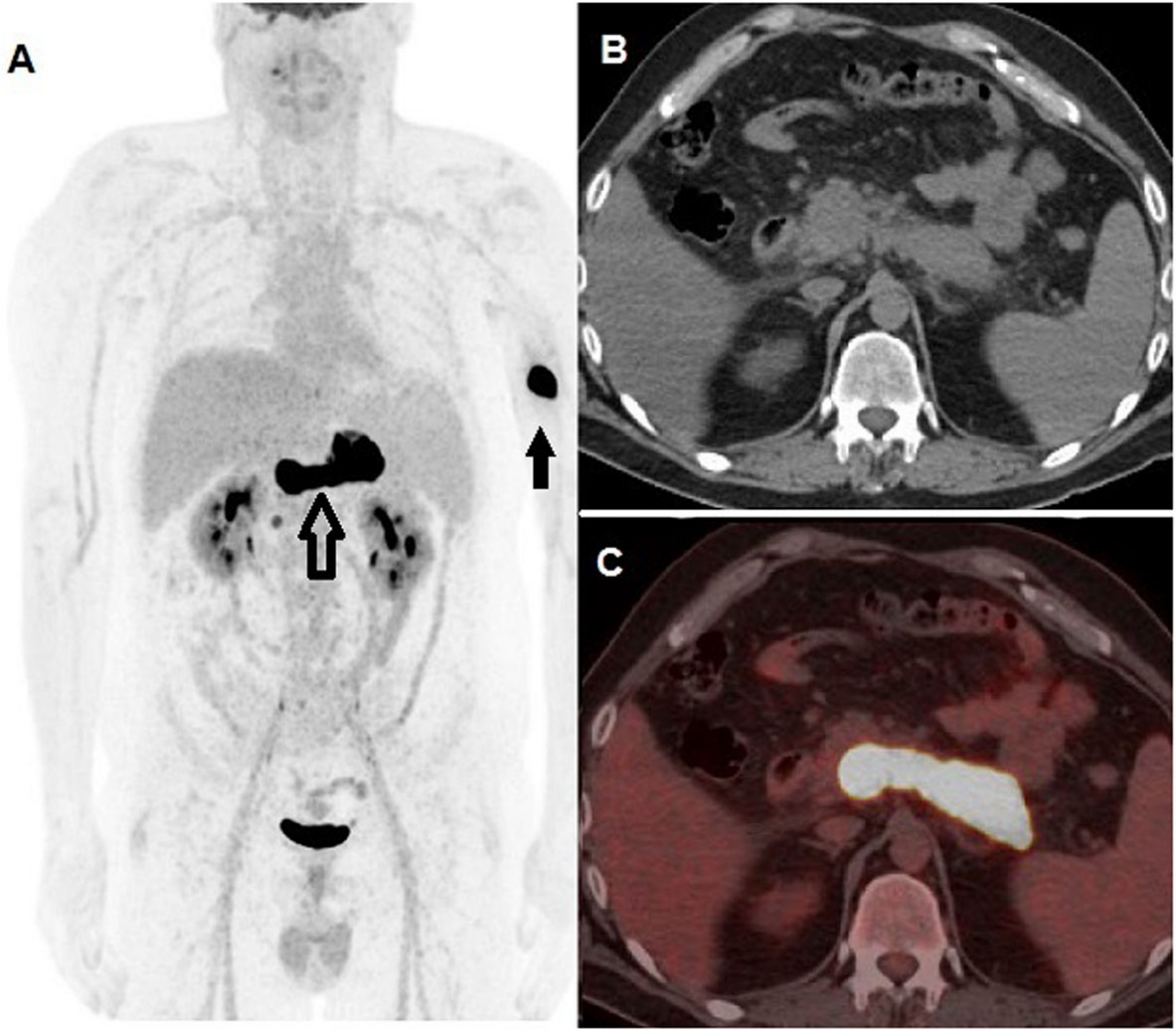
Whole body ^18^F-FDG PET/CT (**A**) demonstrated extremely increased tracer incorporation in the cutaneous/subcutaneous metastasis in the left arm (black arrow) with maximum standardized uptake value (SUVmax) of 67.3 and diffuse and intense ^18^F-FDG uptake in the whole pancreas (SUVmax 99.3, black bordered arrow), which resulted significantly enlarged at the corresponding CT (**B**) and fused PET/CT (**C**) axial slices

**Fig. 2:**
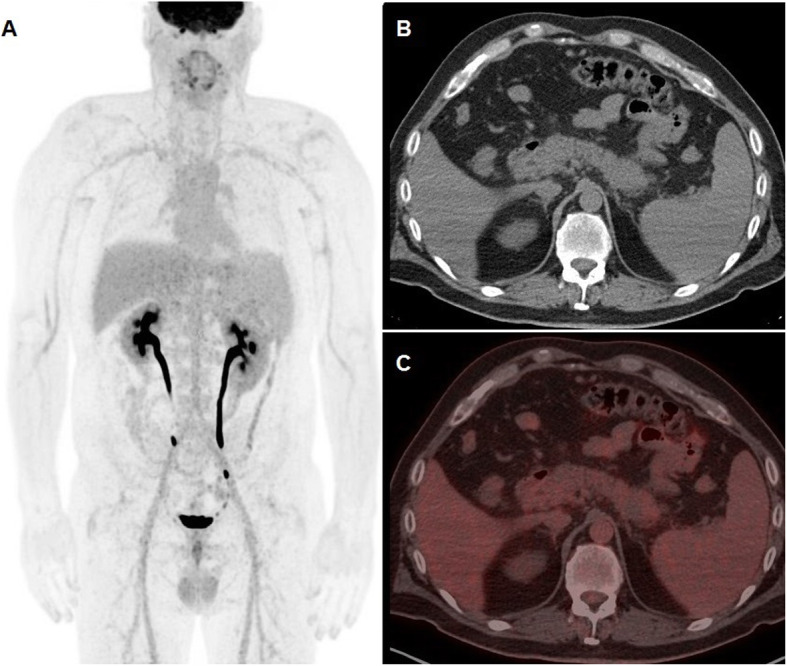
Whole body PET/CT (**A**), CT (**B**) and fused PET/CT (**C**) slices showed complete regression of the hypermetabolic lesions previously described in the left arm and pancreas

Pancreas is a rare localization for MM and, after diagnosis, the sites of primary tumor often remain unknown. Vargas-Jiménez et al. described the case of a 60-year-old man presenting a mass located in the head of the pancreas, showing MRI signals typical for MM metastasis and confirmed by EUS-FNB [1]. A similar report was described by Tma and colleagues, who detected a mass located in the pancreas head in a 71-year-old female, resulted in a metastasis from MM at histology after EUS-FNA [2]. Recently introduced IT through immune checkpoint inhibitors, directed towards specific biomarkers such as programmed death-1 (PD-1) and PD-1 ligand (PDL-1), have thoroughly changed melanoma’s therapeutic landscape [3]. However, it has to be underscored that only 20–40% of patients respond to IT. Therefore, it is of foremost importance to promptly identify subjects who can benefit from such a therapeutic regimen. ^18^F-FDG PET/CT, particularly when specific technical approaches such as dynamic acquisition and analysis of PET-derived parameters are utilized, proved useful to predict MM’s response to IT and offers the unique opportunity to get an insight into tumor’s biology at a molecular level [4-6].

To the best of our knowledge, our case is the first report describing diffuse pancreatic metastatic involvement from MM, evaluated before and after IT through PET/CT imaging. Although a histologic confirmation was not available, the metabolic response of the abdominal lesion to IT, identical to that observed for the histologically confirmed cutaneous lesion, supported the diagnosis of MM pancreatic metastasis.

## Data Availability

Not applicable.
